# Global and 3D Spatial Assessment of Neuroinflammation in Rodent Models of Multiple Sclerosis

**DOI:** 10.1371/journal.pone.0076330

**Published:** 2013-10-04

**Authors:** Shashank Gupta, Regine Utoft, Henrik Hasseldam, Anja Schmidt-Christensen, Tine Dahlbaek Hannibal, Lisbeth Hansen, Nina Fransén-Pettersson, Noopur Agarwal-Gupta, Björn Rozell, Åsa Andersson, Dan Holmberg

**Affiliations:** 1 Department of International Health, Immunology and Microbiology, University of Copenhagen, Copenhagen, Denmark; 2 Department of Experimental Medical Sciences, Immunology, University of Lund, Lund, Sweden; 3 Department of Veterinary Disease Biology, Biomedicine, University of Copenhagen, Frederiksberg C, Denmark; 4 Department of Drug Design and Pharmacology, University of Copenhagen, Copenhagen, Denmark; 5 Department of Biomedical Sciences, BRIC, University of Copenhagen, Copenhagen, Denmark; 6 Department of Veterinary Disease Biology, Pathology, University of Copenhagen, Frederiksberg C, Denmark; 7 Institute of Cellular and Molecular Medicine, University of Copenhagen, Copenhagen, Denmark; Institut Pasteur, France

## Abstract

Multiple Sclerosis (MS) is a progressive autoimmune inflammatory and demyelinating disease of the central nervous system (CNS). T cells play a key role in the progression of neuroinflammation in MS and also in the experimental autoimmune encephalomyelitis (EAE) animal models for the disease. A technology for quantitative and 3 dimensional (3D) spatial assessment of inflammation in this and other CNS inflammatory conditions is much needed. Here we present a procedure for 3D spatial assessment and global quantification of the development of neuroinflammation based on Optical Projection Tomography (OPT). Applying this approach to the analysis of rodent models of MS, we provide global quantitative data of the major inflammatory component as a function of the clinical course. Our data demonstrates a strong correlation between the development and progression of neuroinflammation and clinical disease in several mouse and a rat model of MS refining the information regarding the spatial dynamics of the inflammatory component in EAE. This method provides a powerful tool to investigate the effect of environmental and genetic forces and for assessing the therapeutic effects of drug therapy in animal models of MS and other neuroinflammatory/neurodegenerative disorders.

## Introduction

Multiple sclerosis (MS) is an autoimmune inflammatory demyelinating disorder affecting the central nervous system (CNS) that is characterized by multifocal lesions of the myelinated nerves and clinical signs due to axonal damage [1], [2]. A commonly used animal model for the study of MS is experimental autoimmune encephalomyelitis (EAE) [3]–[5], a model that is used primarily in mice and rats and resembles human MS to varying degrees. The current understanding of the inflammatory and demyelination components of these models has been largely based on immunohistochemical analyses of sections of the affected organs [6] and to magnetic resonance imaging (MRI) studies in mice [7], [8] rats [9], [10] marmoset monkey [11], guinea pigs [12] and humans [13], [14]. Thus, MRI constitutes an important tool to detect CNS atrophy [15], [16] and management of treatment and evaluation of therapeutic effect in MS patients [17]. MRI methodologies have also been very useful in studying EAE progression as they allow direct correlation of histopathological and radiological findings thereby assisting in studying disease pathology [10], Especially Gadolinium enhanced T1W MRI, SPIO and USPIO have been very useful in detecting neuroinflammation in EAE and MS patients [7], [9], [10], [15], [18], [19]. Additionally MRI has been used to quantify inflammation and edema [10], to track macrophages and myelin-reactive T cells [9] as well as to image inflammation in mouse spinal cord during clinical course of EAE progression [8] and to characterize pathological manifestations in different EAE models [20].

While these studies have provided valuable insights into the inflammatory process and the underlying neuro-pathology, they are limited by their levels of resolution and the availability of specific probes. Furthermore, conventional histochemical and immunohistochemical techniques requiring digital reconstruction and extrapolation from 2D sections to generate 3D images have made quantitative analyses of inflammation and neurodegeneration cumbersome and imprecise. Confocal microscopy has so far been the primary option for high-resolution imaging. In this case the limitations have rather been at the level of not allowing global analysis of larger specimens or direct quantification of the processes studied.

The more recent development of optical projection tomography (OPT) [21] has provided a novel imaging platform for the study of autoimmune inflammation in a murine model of type 1 diabetes (T1D). OPT was originally developed for global and 3D spatial assessment of organogenesis in rodent embryogenesis and has been previously applied to quantitate inflammation and β-cell depletion in T1D [22], [23]. Here, we present a protocol to directly measure and visualize the global and 3D development of inflammation in the complete spinal cord and optic nerve of mice and rats with EAE using OPT. This approach provides unprecedented possibilities to globally quantitate and visualize biological process such as inflammation, in the CNS. Thus, in spite of its limitations in being an *ex vivo* technique we anticipate that it will become a powerful tool within neurobiology for the precise spatial analyses of larger specimens of these tissues and constituting a valuable, high resolution imaging complement to in vivo imaging techniques such as MRI.

## Materials and Methods

### Animals

C57BL/6(B6) (H-2^b^) mice were obtained from Taconic laboratories (Denmark) and B10RIII (H-2^r^) mice were bred and kept at the animal house facility at Faculty of Health Sciences, Copenhagen University (SUND-KU). Dark Agouti rats were obtained from the Harlan Lab and housed in the animal facility at SUND-KU. All animal experiments were approved by the animal ethics committee at the University of Copenhagen dnr: 209–561.

### Peptides

Peptides for mouse immunization used were myelin basic protein (MBP) and MOG (myelin oligodendrocyte glycoprotein). MBP_89-101)_: VHFFKNIVTPRTP and MOG_35-55_: MEVGWYRSPFSRVVHLYRNGK were synthesized to >95% purity and obtained from Schafer-N, Denmark.

### Active induction of EAE

For EAE immunizations, mice were anesthetized with isoflurane. C57BL/6 mice were immunized subcutaneously with 150 µg/mouse of MOG_35-55_ peptide, emulsified in complete Freund's adjuvant (CFA) containing 400 µg of Mycobacterium tuberculosis (H37Ra; Difco, Detroit, MI). Additionally, 200 ng of Pertussis toxin (Sigma, St Louis, MO) was given on day 0 and day 2 post-immunization by intraperitoneal injection. Similarly, B10.RIII mice were immunized by intradermal injection with 150 µg/mouse of MBP_89-101_ peptide emulsified in 100 µg of Mycobacterium tuberculosis, with subsequent injection of pertussis toxin (200 ng) on day 0 and day 2. The mice were scored for clinical disease severity. For the immunization of Dark Agouti rats, spinal cords were isolated from the naïve PBS perfused animal, and were immediately homogenized in PBS (33% w/v). CFA emulsion mixture was made in a 1∶1 ratio and each rat was injected with 100 µl subcutaneously, at the base of the tail.

### EAE scoring criteria

Mice were scored and clinically assessed for EAE according to the following scoring criteria as prescribe by the Danish animal ethical committee. (0) no disease, (1) tail paralysis, (2) mild paralysis in one or two hind limbs, (3) moderate hind limb paralysis in one or two limbs, (4) severe paralysis in one or two hind limbs, (5) paralysis in one or two hind limbs and paralysis starting in 4 limbs, (6) moribund. Dark Agouti rats were scored and clinically assessed for EAE to the following scoring criteria (0) no clinical signs of EAE, (1) tail paralysis, (2) paraparesis (3) paraplegia, (4) tetraparesis/paralysis, (5) moribund or dead [24].

### Antibodies

The following primary antibodies were used for labeling CNS specimens for OPT: rabbit anti-CD3 (1∶500, Sigma cat. no. C7930), rabbit anti-MBP (1∶200 Sigma, cat. no M3821), rabbit anti-ASMA (1∶500 Abcam cat. no. ab5694), anti MBP (1∶50, Millipore cat. no. AB9348) and anti F480 (1∶50, Serotec cat. no. MCA497) The secondary antibody was goat anti-rabbit Alexa-594 (1∶500 Invitrogen cat. no. A11012), goat anti-chicken Alexa-488 (1∶3000, Invitrogen cat. no. A11039) and goat anti-rat Alexa-647 (1∶3000, Invitrogen cat. no. A21247).

### Organ preparation, Optical Projection Tomography and global quantification

OPT technology was used to visualize the kinetics of CD3^+^ T cell infiltration in the CNS of EAE rodents. Briefly, mice were deeply anesthetized with isoflurane (Intervet Schering Plough Animal Health) and intracardially perfused with phosphate buffered saline (PBS) followed by 4% paraformaldehyde (PFA, Sigma cat. no. P6148) and then euthanatized. Intact spinal cords and optic nerves were removed and immediately submersed in freshly made 4% PFA in PBS for 3 h at 4°C, then washed in PBS and stepwise transferred to 100% methanol (MeOH) followed by storage at −20°C. These procedures were performed according to previously published protocols for staining, with the exception of our decision to incubate with primary and secondary antibodies for 7 days each. OPT scans were performed using the Bioptonics 3001 OPT M scanner (Bioptonics, Edinburgh, Scotland), with exciter D560/40× and emitter E610lpv2 filter (Chroma) as previously described [23]. Tomographic re-constructions were generated using the NRecon V1.6.1.0 (Skyscan, Belgium) software, and reconstructed images were further assessed using the Bioptonics viewer V2.0 (Bioptonics) as previously described [23]. Final images were made by using either Image J 1.46 or Adobe Photoshop CS3. Quantification of the CD3^+^ T cell infiltrate volumes was performed using 3D image analysis software Volocity v5.2.1 (PerkinElmer) as per published protocol [22] and any possible artifacts were removed manually. Briefly, the CD3^+^ T cell foci volumes were quantified using a measurement protocol created in the quantification software module for volocity. Briefly the image stacks of the tomographic sections were imported into volocity. Thereafter find objects by intensity was applied to the measurement protocol. The protocol picks voxels according to specified intensity threshold values. Intensity threshold values were manually edited to exclude pixels with intensity values lower or higher than those normally contributing the labeled CD3 signals. Thereafter, a fine filter was applied to avoid selection of voxels not contributing to CD3 signals. Potential artifacts such as dust particles were identified in 3D model and removed from the measurement window, to generate volumetric data. The volumetric data were plotted using Graph Pad Prism 6.0 and thereafter correlation analysis was done to calculate the correlation coefficient values (r).

### Cryosectioning

**Figure 1:**
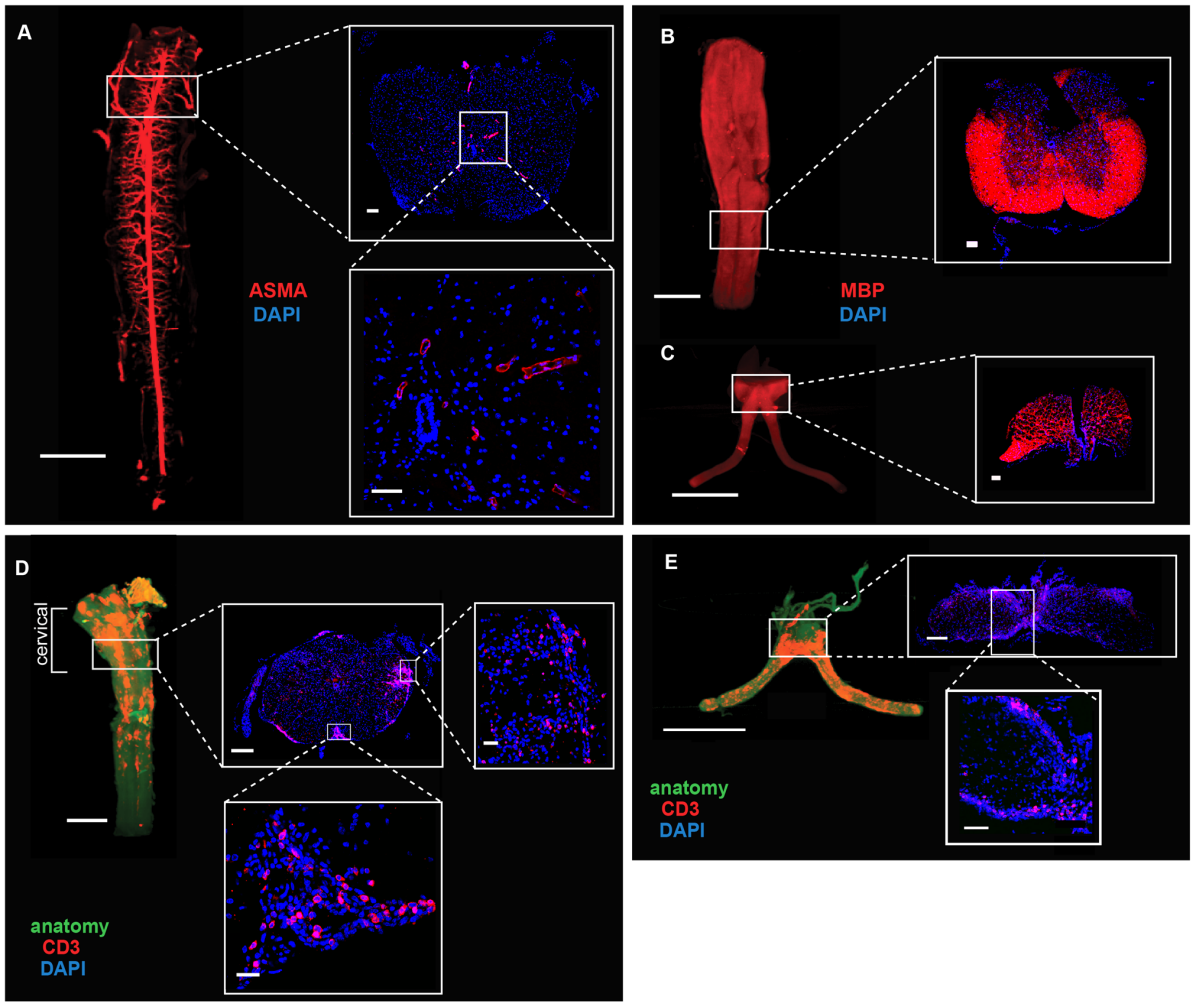
Global and 3D imaging of intact mouse CNS. (A). Spinal cords from C57BL/6 mice were stained with ASMA (red) and were further imaged by OPT. OPT scanned spinal cords were cryosectioned and imaged under a fluorescence microscope with 4× zoom (white frames). Selected regions were also imaged under a confocal microscope with 40× zoom (inlets). Nucleated cells were stained with 4,6-diamidino-2-phenylindole (DAPI). Spinal cord (B) and optic nerve (C) from B10RII mice were stained with MBP (red), and imaged by OPT. Cryosectioning was performed after OPT imaging and images were captured using a fluorescence microscope at 4× zoom. Spinal cords (D) and optic nerves (E) from C57BL/6 mice with a clinical score of 3.0 were stained with CD3 specific antibodies. OPT scanned tissues were cryosectioned and subsequently imaged under a fluorescence microscope at 4× zoom (white frames) or selected regions displaying evidence of CD3^+^ T cell infiltration were imaged using a confocal microscope at 63× zoom (inlets). CD3^+^ T cell staining is shown in red and the anatomy reconstructed from the CNS outline on the basis of the autofluorescence signal is shown in green. Scale bar: (A) Whole organ: 2 mm, 4× zoom images: 100 µm; 40× zoom image: 50 µm; (B, C) 4× zoom image: 100 µm; (D) whole organ: 2 mm, 4× zoom image: 100 µm, 40× zoom images: 50 µm, 63× zoom image: 50 µm; (E) whole organ: 2 mm, 4× zoom image: 100 µm, 63× zoom image: 50 µm.

OPT scanned samples were made BABB (Murray's clear a mixture of benzyl alcohol and benzyl benzoate in ratio 1∶2) free and remaining agarose was trimmed out by washing in 0.29 M sucrose at 57°C. The tissue was then processed for cryosectioning as described previously [25]. 8–10 µm sections were stained with 0.7 µg/ml DAPI without re-staining with specific antibodies for proof of principle studies (Fig. 1). The images were acquired using either an Olympus fluorescence microscope (model BX 43F) with either x4 numerical aperture (N.A) 0.13 or a Leica SP5X confocal microscope equipped with an Apo Fluo x40, N.A. 1.25 oil immersion objective or an x63 water immersion objective with N.A. 1.2. For studying demyelination and F480^+^ cell infiltration the OPT scanned samples were cryosectioned. 8–10 µm sections and were subsequently stained with anti-MBP antibody and anti-F480 antibody. The images were acquired using Carl Zeiss Imager.M2 AXIO equipped with a Plan-Apochromat x20, N.A. 0.8. Images were captured using tile scan function in either 4×4 or 5×5 tile mode and then were stitched together using inbuilt ZEN 2012 software to generate intact high resolution images of the sections.

## Results and Discussion

### Global and 3D imaging of mouse CNS by OPT

To investigate whether OPT [21] could be applied to global quantification and 3D imaging of the adult mouse CNS, we isolated spinal cords from C57BL/6 (B6) and B10.RIII mice and stained them with antibodies specific for antigens expressed by various CNS tissues. As illustrated in Fig. 1A and Movie S1, the larger blood vessels were visualized in the intact mouse spinal cord by staining with anti-smooth muscle actin (ASMA) antibodies and submitting the specimens for OPT. Similarly, myelin basic protein (MBP)-expressing spinal cord (Fig. 1B) and optic nerve tissue (Fig. 1C) were globally visualized by staining with anti-MBP antibodies. We next investigated whether OPT could also be used for global imaging of neuroinflammation. For this purpose, EAE was induced in B6 mice by immunizing with MOG_35-55_ peptides [26] and the spinal cords and optic nerves of mice having a clinical score of 3.0 (moderate hind limb paralysis) were stained with anti-CD3 antibodies to reveal T cell infiltration. As shown (Fig. 1D-E) specific CD3 staining was observed throughout the entire inflamed spinal cord and optic nerve tissue obtained from EAE mice. The overall distribution of CD3 staining suggested that antibodies had penetrated the entire tissue. This was more clearly visualized in a 3D video recording of the scanned specimen as illustrated in Movie S2 (spinal cord) and Movie S3 (optic nerve). A general concern when applying imaging modalities based on specific antibodies to study specimens of CNS origin is the limitations in penetration depth of the antibody [8]. For this reason we followed up the OPT scanning of the spinal cords and optic nerves by sectioning the scanned specimens and inspecting them in conventional fluorescence microscopy and confocal microscopy without re-staining. As illustrated in Fig. 1, ASMA and anti-CD3 were found to stain cells with the expected distributions in the tissues and correlated well with the staining pattern obtained by OPT scanning. Similarly, the pattern of anti-MBP staining of sections of the spinal cord and the optic nerve displayed the expected distribution and correlated with the OPT data obtained from the same specimens. We could in this way confirm that the samples prepared according to the OPT staining protocol displayed full penetration of the specific antibodies and were indeed reflecting specific cellular staining corresponding to the infiltrating CD3^+^ T cells.

Previously used methods to visualize the neuroinflammation have been mainly based on conventional immunohistological techniques with limited ability to capture the process in a global and 3D fashion [6], [27], [28]. Here we could demonstrate that optical tomography imaging, in unique way, could be used to image rodent CNS providing detailed 3D spatial data of the development of inflammation in the spinal cord and optic nerves during the clinical course of EAE. In contrast to immunohistological technique our method allows for imaging of the intact mouse spinal cord and optic nerves giving a comprehensive picture of the disease process globally.

Because some animal models of EAE display inflammation not only in the spinal cord and optic nerve but also in the brain [7], we also tested if OPT could be used to quantitate inflammation at this site. In this case, however, we were not been able to score infiltrating CD3^+^ cells further than 1–2 mm from the surface of the organ (data not shown). It is possible that with further optimization of the protocol for staining and clearing of the specimen this caveats could be overcome. The brain being inaccessible to efficient OPT scanning of inflammation restricted our studies to the study of the spinal cord and optic nerve. Nevertheless, we were successful in imaging the intact spinal cord and optic nerves from the mouse CNS.

### Imaging progression of neuroinflammation and demyelination

**Figure 2:**
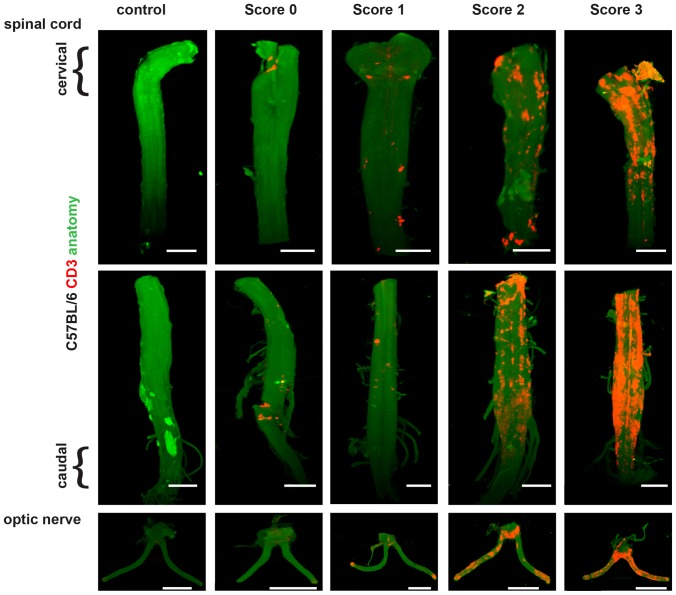
Spatial assessment of neuroinflammation in B6 EAE. C57BL/6 mice were immunized with MOG_35-55_/complete Freunds adjuvants (CFA)/pertussis toxin (PTX) and were observed for EAE. CNS tissue was obtained at different clinical scores and was stained for CD3^+^ T cells and imaged using OPT. The iso-surface rendered OPT images of spinal cord and optic nerve sections at different clinical scores during the progression of EAE were obtained. Infiltrating CD3^+^ T cells (red) due to the signal from the CD3 specific antibody. The reconstruction of the CNS outline is based on the autofluorescence signal (green), where the upper part of spinal cord is the cervical and thoracic region while the bottom parts are the lumbar and sacral regions. Images were generated for score 1 (n = 2), score 2 (n = 3), score 3 (n = 2). Scale bar = 2 mm.

**Figure 3:**
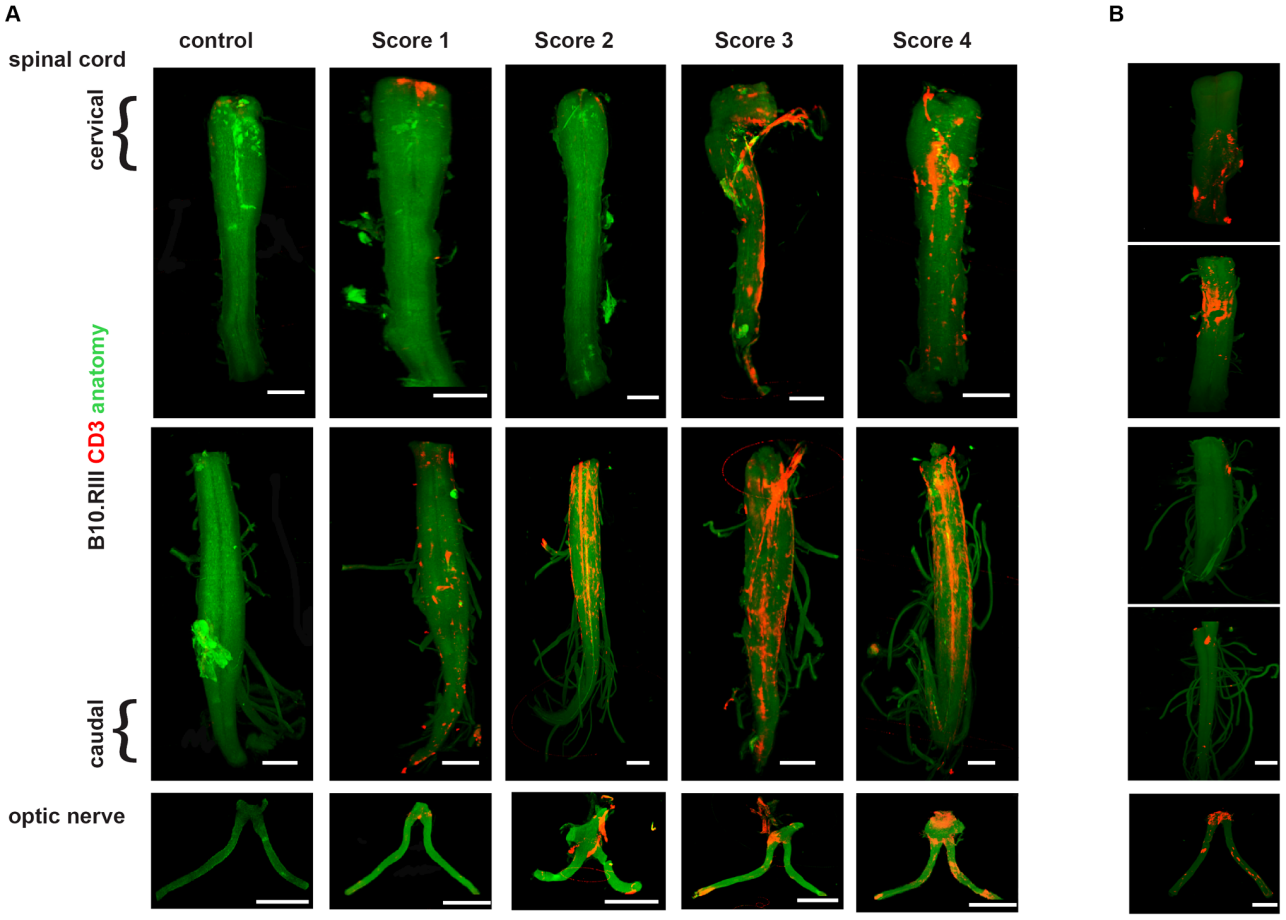
Spatial assessment of neuroinflammation in B10.RIII EAE and DA Rat EAE model. (A) B10.RIII mice were immunized with MBP_89-101_/CFA/PTX and were EAE was allowed to progress. At different clinical scores CNS tissue was obtained and was stained for CD3^+^ T cells and imaged using OPT. The iso-surface rendered OPT images of spinal cord and optic nerve sections at different clinical scores during the progression of EAE were obtained. Infiltrating CD3^+^ T cells (red) due to the signal from the CD3 specific antibody. The reconstruction of the CNS outline is based on the autofluorescence signal (green), where the upper parts of spinal cord are the cervical and thoracic region while the bottom parts are the lumbar and sacral region. Images were generated for score 1 (n = 4), score 2 (n = 5), score 3 (n = 3) score 4 (n = 4). (B) Intact spinal cord and optic nerves were removed from Dark Agouti rats with a clinical score of 4, and then stained with anti-CD3 antibody. Global imaging was performed using OPT. Iso-surface rendered OPT images of representative spinal cords and optic nerves were obtained. Infiltrating CD3^+^ T cell lymphocytes staining (red) are based on the signal from the CD3 specific antibody. The reconstruction of the CNS outline is based on the autofluorescence signal (green). Scale bar = 2 mm.

To image the progression of neuroinflammation, B6 mice were immunized with MOG_35-55_/CFA and their clinical scores were recorded. Mice were sacrificed when they reached defined clinical scores (clinical scores 1–4). The spinal cords and optic nerves were collected and stained with anti-CD3 antibodies to reveal infiltrating T cells and then monitored by OPT. In this manner, it was possible to assess the progression of infiltrating CD3^+^ T cells in the spinal cords and optic nerves of diseased mice (Fig. 2 and Movie S2 and S3). The progression of CD3^+^ T cell infiltration paralleled the increase in the clinical score. At a score of 2 or 3, inflammatory foci were observed in the spinal cord with significant T cell infiltration. Inflammation was observed in both ends of the optic nerve, at the retina and at the optic chiasm. Similar results were obtained when the OPT imaging protocol was applied to the analysis of another mouse model of EAE, in which B10.RIII mice were immunized with MBP_89-101_/CFA [29] (Fig. 3A). Also in this case spinal cords and optic nerves were collected and stained with anti-CD3 antibodies and then monitored by OPT to reveal infiltrating T cells. Similar to what was observed in the EAE B6/MOG_35-55_ model, we found a direct correlation between the clinical score and the amount of CD3^+^ T cell infiltration, with intense staining of CD3^+^ T cells observed in the spinal cord and the optic nerve. This is in line with previous published results showing increase in CD4^+^ T cells [27], [30], with increasing clinical score during the clinical progression of EAE. Time course of the pathological events during EAE have been shown previously [6], [27], [28], primarily in the sections. Here we show an OPT based method to visualize ongoing inflammation in the intact CNS globally, in particular with CD3^+^ T cell infiltration. Recently we were able to image transplanted islets in human liver from T1D patient autopsy and healthy pancreas by employing OPT [31], which opens up the possibilities of imaging human CNS obtained from MS autopsies in near future.

**Figure 4:**
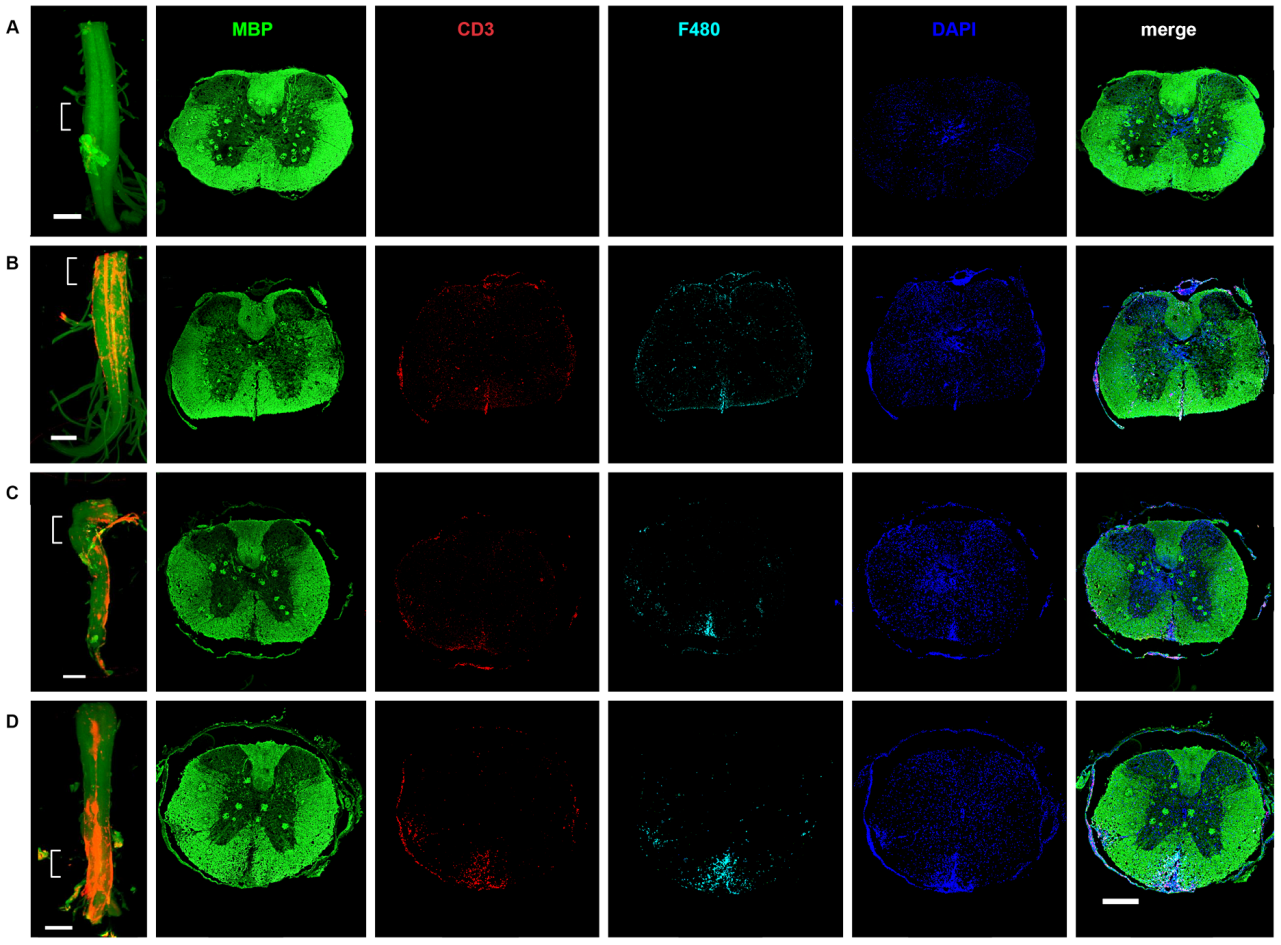
Progression of inflammation and demyelination during clinical course of EAE in B10.RIII EAE model. OPT scanned samples were subjected to cryosectioning and subsequently sections were stained with anti-MBP (green) and anti-F480 (cyan). CD3 staining visualizing T cells came from the pre-stained OPT samples. Spinal cord sections obtained from mice with different clinical scores were stained and imaged to record inflammation and demyelination during the progression of EAE. No inflammation was observed in the control spinal cord section (A). Increase in loss of myelin, F480^+^ cells infiltration and CD3 ^+^ T cell infiltration was observed with increase in clinical score severity as shown for score 2 (B) score 3 (C) and score 4 (D). Images were captured at 20× magnification. Sections roughly come from the indicated areas of the intact spinal cord. Data was generated mice/group (n = 2). Scale bar = 500 µm.

OPT scanned samples can be further sectioned and re-stained with different antibodies to reveal detailed information of the cellular infiltration [25]. To get a detailed cellular insight into and compare the progression of inflammation and demyelination, OPT scanned B10.RIII EAE samples were further subjected to de-embedding followed by cryosectioning. Sections were stained with anti-F480 to visualize macrophage infiltration and anti-MBP to visualize loss of myelin. CD3 staining visualizing T cells came from the pre-stained OPT samples. Spinal cord from control mice showed no demyelination with intact MBP staining shown in green (Fig. 4A). MBP immunoreactivity was also comparatively less affected in EAE mice with score 1 (data not shown). Mice with clinical score 2 showed deterioration in myelin. Increase in the loss of myelin was seen with increase in the clinical score (Fig. 4B). Mice with clinical scores 3 and 4 showed pronounced loss of myelin with demyelinated plaques (Fig. 4C & 4D). Similarly a high number of cellular infiltration was observed in the spinal cord as shown by DAPI staining (blue). The recruitment of F480^+^ macrophages (cyan) and CD3^+^ T cells (red) to CNS observed to be increased with EAE severity (Fig. 4). This is in line with previous published data showing increase in CD4^+^ T cells [27], [30], demyelination [32], and lesion size [28] with increasing EAE severity. Overall we observed a parallel increase in demyelination, F480^+^ macrophage and CD3^+^ T cell infiltration in the CNS with an increase in EAE severity.

Finally, we tested whether OPT could also be applied to image rat CNS. Therefore dark agouti (DA) rats were immunized with spinal cord homogenate/CFA to induce active EAE [33]. Rats were sacrificed when the clinical score reached 4; spinal cord and optic nerve tissue were obtained and stained for CD3^+^ T cells and then imaged by OPT. As illustrated in Fig. 3B, a significant number of CD3^+^ T cells in the spinal cord as well as in the optic nerve were visualized in the DA model of EAE. Spinal cords and optic nerve with a clinical score of 2 were also imaged by OPT and there relatively less CD3^+^ T cell infiltration was observed (data not shown) as compared to spinal cord with clinical score 4.

### Quantification of inflammation

**Figure 5:**
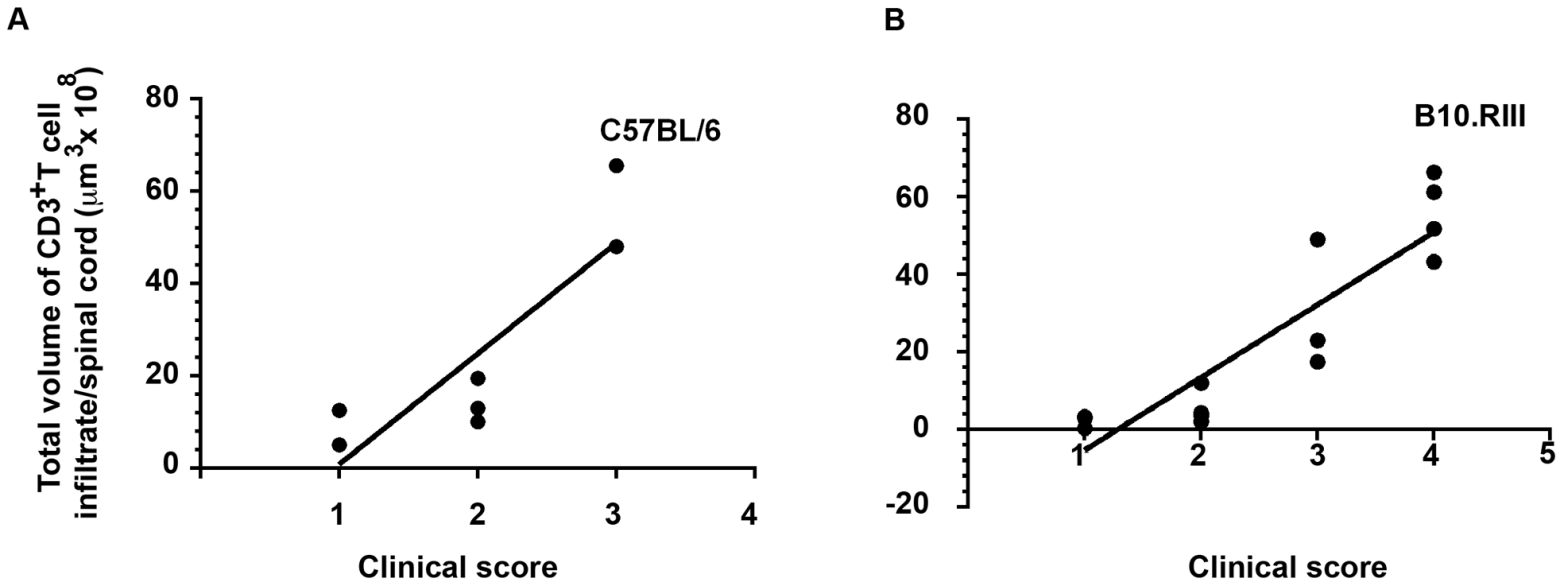
Global quantification of the CD3^+^T cell inflammatory foci in EAE. B6/MOG (A) and B10RIII/MBP (B) models were analyzed. The total volume estimates of CD3^+^ T cell infiltration was measured by OPT and calculated using Volocity software. Each bar indicates the results from an individual spinal cord. The Y-axis indicates the infiltrated CD3^+^ T cell volumes/spinal cord, and the X-axis indicates the clinical score. Correlation analysis between the volumes of CD3^+^ T cell foci and corresponding clinical scores in B6 mice r = 0.90513) (Fig. 5A) and B10.RIII r = 0.898869 (Fig. 5B). Each dot in the correlation plot represents one mouse. For B6 EAE quantification mice numbers were: score 1 (n = 2), score 2 (n = 3), score 3 (n = 2) while for B10.RIII quantification mice numbers were: score 1 (n = 4), score 2 (n = 5), score 3 (n = 3) score 4 (n = 4).

After establishing that the OPT imaging approach could be used to monitor ongoing inflammation in the CNS, we tested if this approach could be used to directly quantify the inflammatory process. To globally quantify inflammatory foci during the progression of EAE in mice (Fig. 2 and Fig. 3A), we used Volocity 3D image analysis software to calculate the total volume of CD3^+^ T cell infiltration in the affected spinal cords of the OPT scanned samples in B6 (Fig. 5A) and B10.RIII (Fig. 5B) EAE models. Thereafter a correlation analysis was performed between the clinical score and volume of CD3^+^ T cell infiltration in the spinal cords of B6 model and B10.RIII model. Strong correlation between the total volumes of the foci with infiltrated CD3^+^ T cells to the clinical score was observed during the progression of CD3^+^ T cell infiltration into the CNS in B6 EAE model with r = 0.905123 (Fig. 5A) and B10.RII EAE model with r = 0.898869 (Fig. 5B). Clinical score dependent increase in CD4^+^ T cells has been shown before by research groups analyzing the CNS tissue from EAE affected mice [27], [30].

## Conclusions

In the present study we evaluated the OPT imaging approach for the study of inflammatory and demyelinative processes of the CNS and to use this technique to quantitate these processes. We could demonstrate that optical tomography imaging, in unique way, could not only provide detailed 3D spatial data of the development of inflammation, and demyelination in the spinal cord and optic nerves but also could be used to directly quantify this process. While the findings reported here focused on monitoring the CD3^+^ T cell component of the inflammatory process, we anticipate that employing markers for other cellular subsets in similar studies may reveal the contribution of different cell types in the development and progression of neuroinflammation. Thus, in spite of the obvious limitations in OPT that lies in the fact that it is an *ex vivo* technology, this method has significant potential to become a powerful tool for visualizing various forms of disease processes involving the CNS both in animal models and in autopsy material from patients.

## Supporting Information

Movie S1 **3D visualization of ASMA stained vessels in a mouse spinal cord.** Iso-surface reconstruction of a spinal cord from C57BL/6 mice stained with ASMA. The reconstruction of the spinal cord outline is based on autofluorescence signal (green) whereas ASMA labeled blood vessels are visualized in red. Scale bar = 2 mm.
(AVI)

Movie S2 **3D visualization of CD3^+^ T cell infiltration in a EAE inflamed mouse spinal cord.** CD3 stained Iso-surface reconstruction of a spinal cord obtained from EAE affected B6 mouse with a clinical score of 3. The reconstruction of the spinal cord outline is based on autofluorescence signal (green) whereas CD3^+^ T cells are visualized in red. Scale bar = 2 mm.
(AVI)

Movie S3 **3D visualization of CD3^+^ T cell infiltration in a EAE inflamed mouse optic nerve.** Iso-surface reconstruction of an optic nerve obtained from EAE affected B6 mouse with a clinical score of 3. The reconstruction of the spinal cord outline is based on autofluorescence signal (green) whereas CD3^+^ T cells are visualized in red. Scale bar = 2 mm.
(AVI)
